# S78. Proffered paper: High-affinity CD20-specific TCRs suitable for adoptive immunotherapy can be readily isolated from the allo-repertoire using reverse immunology

**DOI:** 10.1186/2051-1426-2-S2-I16

**Published:** 2014-03-12

**Authors:** L Jahn, P Hombrink, C Hassan, MGD Kester, MP Schoonakker, JHF Falkenburg, P van Veelen, MHM Heemskerk

**Affiliations:** 1 Leiden University Medical Center, Hematology, Leiden, the Netherlands; 2 Sanquin Research, Hematopoiesis, Amsterdam, the Netherlands; 3 Leiden University Medical Center, Immunohematology and Blood Transfusion, Leiden, the Netherlands

## 

Studies using T-cell receptor (TCR) or chimeric antigen receptor (CAR) transduced T-cells have shown the effectiveness of adoptive immunotherapy to treat different malignancies. The efficacy and safety of such interventions greatly depends on good target selection to prevent on-target toxicity. Furthermore, the broad application of TCR-based adoptive immunotherapy is hampered by a lack of an effective immune response against self-antigens. Through self-tolerance, T-cells carrying high-affinity TCRs reactive to self-antigens are deleted during thymic selection. An attractive strategy is to exploit the immunogenicity of foreign human leukocyte antigen (HLA) molecules to generate an effective immune response against these antigens. Here, we describe a protocol to efficiently isolate high-avidity alloHLA-restricted T-cells targeting the B-cell compartment.

From a B-cell peptide elution library 15 peptides derived from genes exhibiting B-cell restricted expression patterns were identified and peptide-MHC multimers (pMHC) of HLA-A*0201 were generated. Via MACSorting and FACSorting a plethora of pMHC-multimer binding T-cell clones from HLA-A*0201-negative individuals were isolated. Generated T-cell clones were selected based on peptide-specificity and avidity for further characterization.

We successfully isolated two distinct T-cell clones carrying high-affinity TCRs specific for a CD20 peptide presented in HLA-A*0201. CD20 dependent recognition could be demonstrated by genetically engineering CD20-negative K562-A2 cells to express CD20. The isolated T-cell clones efficiently recognized CD20-expressing HLA-A*0201 primary chronic lymphocytic leukaemia (CLL), acute lymphoblastic leukaemia (ALL) and mantle cell lymphoma (MCL), while recognition of CD20-negative hematopoietic and non-hematopoietic cell-subsets was absent. In addition, the CD20-specific T-cell clones were able to more efficiently recognise ALL cell-lines than CD20 specific antibodies. We demonstrated that on ALL cell lines with only very low CD20 surface expression, the CD20-specific T cell clones could still efficiently recognise endogenously processed CD20-derived peptides in the context of HLA-A*0201.

In conclusion, we developed a platform for the rapid identification of high-affinity TCRs of therapeutic relevance targeted to self-antigens by combining gene expression data with valuable information on peptide processing from peptide elution studies and exploiting the immunogenicity of foreign HLA. Using this platform we successfully isolated CD20-specific TCRs which could broaden the application of immunotherapies targeted to CD20 in cases were CD20-cell surface expression is low. Based on its general principle the developed platform could easily be adapted to target other malignancies.

